# Stem Cells and Organoid Technology in Precision Medicine in Inflammation: Are We There Yet?

**DOI:** 10.3389/fimmu.2020.573562

**Published:** 2020-12-21

**Authors:** Florian Tran, Christine Klein, Alexander Arlt, Simon Imm, Evelyn Knappe, Alison Simmons, Philip Rosenstiel, Philip Seibler

**Affiliations:** ^1^Institute of Clinical Molecular Biology, University of Kiel, Kiel, Germany; ^2^Klinik für Innere Medizin I, Universitätsklinikum Schleswig-Holstein, Kiel, Germany; ^3^Institute of Neurogenetics, University of Lübeck, Lübeck, Germany; ^4^University Department for Gastroenterology, Klinikum Oldenburg AöR, European Medical School (EMS), Oldenburg, Germany; ^5^MRC Human Immunology Unit (MRC), University of Oxford, Oxford, United Kingdom; ^6^Translational Gastroenterology Unit, University of Oxford, Oxford, United Kingdom

**Keywords:** stem cell, cancer, precision medicine, co-culture, host-microbe, immune-epithelial interactions, induced pluripotent stem cells, patient derived organoids

## Abstract

Individualised cellular models of disease are a key tool for precision medicine to recapitulate chronic inflammatory processes. Organoid models can be derived from induced pluripotent stem cells (iPSCs) or from primary stem cells *ex vivo*. These models have been emerging over the past decade and have been used to reconstruct the respective organ-specific physiology and pathology, at an unsurpassed depth. In cancer research, patient-derived cancer organoids opened new perspectives in predicting therapy response and provided novel insights into tumour biology. In precision medicine of chronic inflammatory disorders, stem-cell based organoid models are currently being evaluated in pre-clinical pharmacodynamic studies (clinical studies in a dish) and are employed in clinical studies, e.g., by re-transplanting autologous epithelial organoids to re-establish intestinal barrier integrity. A particularly exciting feature of iPSC systems is their ability to provide insights into organ systems and inflammatory disease processes, which cannot be monitored with clinical biopsies, such as immune reactions in neurodegenerative disorders. Refinement of differentiation protocols, and next-generation co-culturing methods, aimed at generating self-organised, complex tissues *in vitro*, will be the next logical steps. In this mini-review, we critically discuss the current state-of-the-art stem cell and organoid technologies, as well as their future impact, potential and promises in combating immune-mediated chronic diseases.

## Stem Cells and Organoids – The Rise of Novel Model Systems

**Figure 1:**
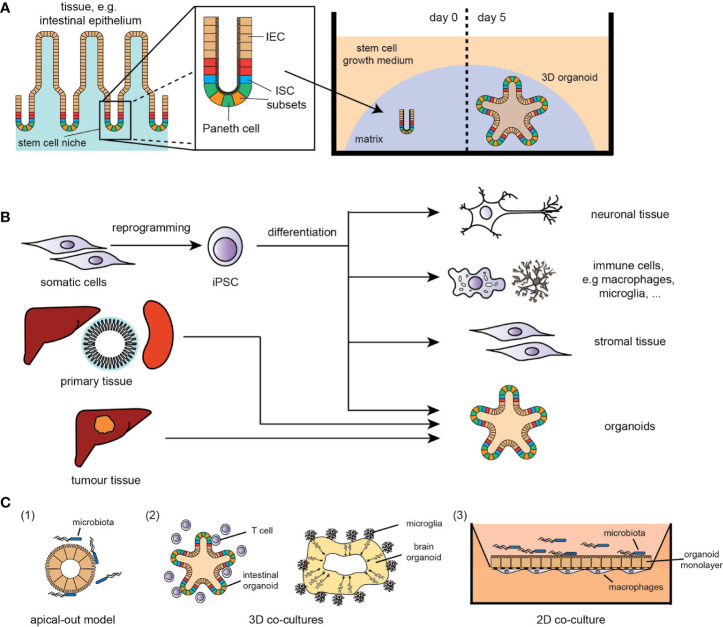
**(A)** Example of adult tissue-derived organoids (here: small intestine); IEC, intestinal epithelial cells; ISC, intestinal stem cell. **(B)** Overview of stem cell- and (tumour) tissue-derived *in vitro* models (selection); iPSC, induced pluripotent stem cells. **(C)** Overview of co-culture systems (simplified selection): (1) apical-out model of intestinal organoids for host-microbe-interaction studies, (2) 3D co-culture systems of immune cells with organoids, e.g., T cells/intestinal organoids and microglia/brain organoids, (3) 2D co-culture systems with a polarized organoid monolayer with either immune cell (basal interface) or microbe (apical interface).

Chronic inflammatory diseases are characterised by either a persisting stimulus for inflammatory signals and/or an inadequate resolution of a response to tissue insults, leading to the development of cancer, neurodegenerative or autoinflammatory disorders. In this complex, disharmonised multi-cellular immune response, the epithelium, immune cells and the microbiome play central roles and thus the development of adequate *in vitro* models of disease, reflecting the complexity of immune interactions in chronic inflammation, is an urgent need in all biomedical fields. However, tissue biology is very challenging to study in mammals, and progress can be hindered by sample accessibility and ethical concerns in humans. One of the cardinal concepts underlying organoid technology is the idea that stem cells have the intrinsic ability to self-organise into 3D structures that resemble *in vivo* organs. A major breakthrough was achieved in 2009, when adult tissue-resident stem cells were found to proliferate and self-organise, *in vitro*, into organoids (1, 2) (**Figure 1A**). The method has since been adapted to generate murine and human organoids from epithelial tissues of major organs like skin, kidney, liver and intestine (3–6) (**Figure 1B**), and used for various physiological and disease-related studies, e.g. for complex disorders like inflammatory bowel disease (IBD). Using intestinal organoids, the functional link between the IBD risk gene *ATG16L1*, interleukin(IL)-22 signalling and STING-dependent type-I-interferon (IFN-I) response was discovered (7). Similarly, the role of the DNA damage repair gene *RNASEH2B* in intestinal inflammation and tissue regeneration was identified in organoid models (8). Purified intestinal epithelium from inflamed intestinal tissue obtained from IBD patients displayed distinct epigenetic and transcriptional alternations, which were retained in organoid cultures and correlated with disease outcome (9). Inflamed IBD tissue-derived organoids display increased activation of typical molecular hallmarks of IBD such as the ATF6 pathway as a branch of the unfolded protein response (UPR), which were used to identify novel anti-inflammatory ATF6 targeting compounds (10). As organoids can provide experimental manipulability and maintain at the same time biologic complexity, they function as a bridge between conventional 2D cell culture and animal models (2).

A further breakthrough was the development of human induced pluripotent stem cell (iPSC)-derived organoid culture. Intestinal organoids were one of the first iPSC-derived tissues consisting of a polarised, columnar epithelium patterned into villus-like structures and crypt-like proliferative zones (11). The iPSC technology has the enormous potential to overcome the limitations of accessibility of specific tissues, such as brain or heart, providing improved patient-derived cellular models of human disease that can also be used for drug screens and personalised treatment strategies (**Figure 1B**). Cerebral brain organoids displayed various discrete though interdependent brain regions (12). This potential is further strengthened by combining iPSC technology with genome engineering - allowing the correction of mutations in patient-derived iPSCs (13) and the modification of reporter lines, thereby facilitating differentiation towards specific cell types (14).

In the following, we will discuss the technical progress and remaining limitations on using more complex cell culture models to understand inflammatory disorders, host–pathogen interactions or cancer.

## Precise Disease Modelling: Co-Cultures and Tissue Engineering to Study Autoimmune Diseases and Infections

A wide range of co-culture and organoid-based disease models that reproduce genetic immune diseases (7, 8, 15) and host–pathogen interactions (16, 17) have already been developed and provide proof of principle that these complex cultures can show certain well-known pathological features. These model systems are the result of tremendous advances and bioengineering innovations of the past decade.

Impaired epithelial host-microbe interaction is one of the important features of IBD and thus, model systems focusing on this interplay are needed (18). Due to the “apical-in/basolateral-out” polarity of conventional organoid systems, microbes need to be microinjected into the lumen of the organoids to mimic physiological host-microbial cross-talks (19–21). This technique however is slow, even with standardised high-throughput approaches technically very challenging and the reproducibility of the results is low due to the heterogeneity of the organoids and their luminal content (22).

To combat this issue of “wrong” epithelial polarity, an “apical-out” system with reverse organoid polarity was developed (22) (**Figure 1C**), providing a more suitable and reliable model for processes and properties of the epithelium, e.g. examining nutrient absorption and host-pathogen interactions, such as inflammatory cytokines/chemokines, antimicrobial peptides and ROS production.

Another approach to assess host-pathogen interactions is the co-culturing of an epithelial monolayer of preserved apical-basolateral polarity with selected pathogens (**Figure 1C**). Using such models, the role of *K. pneumoniae* in priming T_H_17 response in primary sclerosing cholangitis (15) or the pathophysiology of chronic *Helicobacter pylori* infections (20, 23) were identified. Recently, microfluidic-based systems, i.e. the *human microbial crosstalk* (HuMiX) system (24, 25), were established, enabling co-culturing of human intestinal epithelial monolayers with anaerobic pathogens and the analysis of diet-microbiome-human interactions. Besides studies on antimicrobial response, the development of two-dimensional organoid cultures with an air-liquid-interface increased the complexity and maturation of the epithelial layer, providing a better approximation to the *in vivo* tissue, similar to colonic monolayers (26), and led to the discovery of a novel injury-related cell type, associated with IBD-related tissue regeneration.

The interaction of the epithelium with the stroma, including immune cells, mesenchymal cells and neuronal cells, is vital for organ development and homeostasis and needs to be taken into account when modelling complex immunological diseases like IBD (27). Stromal cells provide the niche for stem cell growth by production of ECM and secretion of essential growth factors, such as WNT proteins (27) and R-spondin (28). Various *in vitro* models (both 2D and 3D) have been co-cultured with different types of immune cells to assess immune-related orchestration of the epithelial barrier and vice versa the communication back to the specialised immune cells (29). E.g., co-culturing of intestinal organoids and macrophages led to enhanced barrier integrity (30), and the epithelial-macrophage communication enabled a more coordinated immune response to infection.

The enteric nervous system (ENS), controlling several functions, such as motility and permeability, has been linked to enteric neuropathies and gut disorders (31). To that end, a model of co-culturing of intestinal epithelial cells with ENS neurons and glia or subepithelial myofibroblasts was developed (32), showing that the ENS influences intestinal stem cell (ISC) fate by increasing differentiation towards the enteroendocrine lineage and thereby modulates intestinal barrier function. Vice versa, immune cells like tissue resident macrophages upon gastrointestinal infections modulate cellular fate of enteric neurons (33). Going one step further, human iPSC-derived intestinal tissue with a functional ENS was generated by combining intestinal organoids with neural crest cells, in order to investigate the pathophysiology of *Hirschsprung’s disease* (31). This model could also provide a platform for analysing the epithelium/ENS-axis in IBD.

Vice versa, environmental factors influencing the microbiome (e.g. intestinal infections) are suggested to play a key role in the initiation and progression of neurological disorders, such as Parkinson’s disease (PD) (34–36). PD is characterised by the loss of dopaminergic neurons and intracellular inclusions composed mainly of alpha synuclein, which can be identified in intestinal biopsies years prior to PD diagnosis (37). Notably, it was shown that inflammatory processes, e.g. mitochondrial stress-associated STING/IFN-I response in the absence of PD-linked Parkin or Pink1 function, are drivers of PD pathology (36, 38). Therefore, it is of interest not only to examine disease mechanisms of the gut-brain axis but also direct inflammatory processes in the brain triggered by microglia activation (39). In this regard, brain organoids in co-culture with microglia represent a major breakthrough in neuronal disease modelling techniques (40) and will serve to facilitate the development of more precise human brain models for basic mechanistic studies in neural-glial interactions and drug discovery.

There remains one crucial factor none of these models accounts for, that is blood vessels. Aside from transporting the vital contents of our blood to and from the demanding organs, vascularization is a critical component of physiological (and pathological) development. Recently, human pluripotent stem cells were engineered to induce endothelium development by expressing ETS variant 2, which contributed to forming a complex vascular-like network in human cortical organoids (41). Vascularized organoids displayed enhanced functional maturation and acquired several blood-brain barrier characteristics, including an increase in the expression of tight junctions, nutrient transporters and trans-endothelial electrical resistance. These cultures formed vasculature-like structures that resemble the status in early prenatal brain and present a robust model to study disease *in vitro*.

The key challenge of the next years will be the establishment of multi-compartment *in vitro* models for disease and beyond the two-compartment organoid co-culturing systems [e.g. organ/body-on-a-chip, including tissue cultures from multiple organs (42, 43)], to really represent complex, multi-organ immunological disorders.

## Patient in a Dish: Excursus on Predicting Therapy Response in Inflammation-Driven Cancer

**Figure 2:**
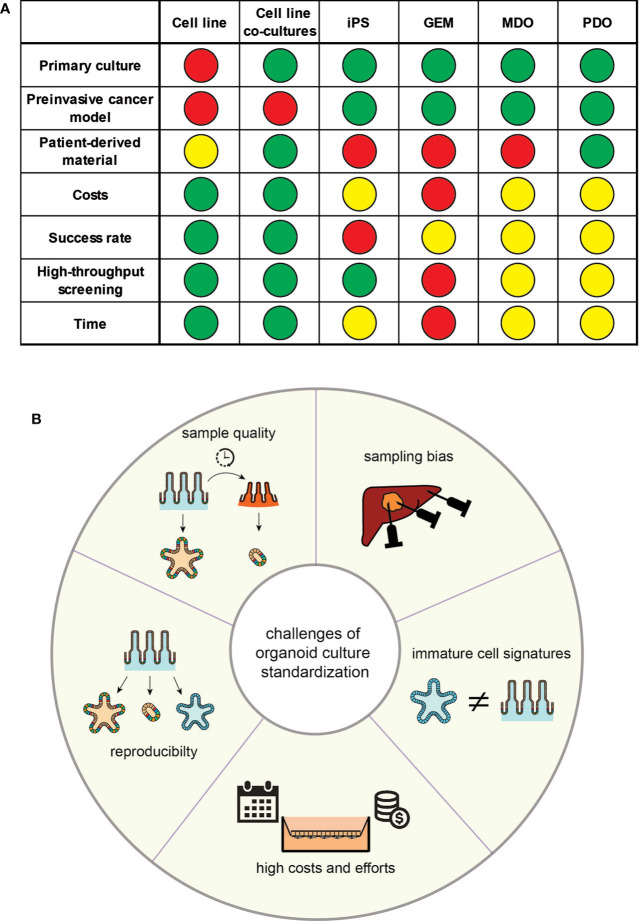
**(A)** Properties of the discussed models for translational research; GEM, genetically engineered mouse model; MDO, murine-derived organoid; iPS, induced pluripotential stem cell; PDO, patient-derived organoid; definition of colours: Green - possible/cheap (costs)/easy (success rate)/1–2 weeks (time); Yellow: medium (costs)/medium (success rate)/1–2 months (time); orange – 1–6 months (time); red - difficult/expansive (costs)/low (success rate)/over 6 months (time). **(B)** Overview over the proposed challenges in the standardisation of organoid culturing systems.

Inflammation is a driving aspect of oncogenesis and a necessary component of the established tumour’s mechanism of resistance. This aspect of the immune-oncological crosstalk has recently gained attention due to the use of immunotherapy in nearly all type of cancer (44). Previous oversimplified cell culturing methods lack the tumour’s micro-environment, which have only been partially resolved by co-culturing models of tumour cell lines with other somatic cells (45–47) (**Figure 2A**). Animal models, including genetic tumour models (GEM), murine-derived organoids (MDO) and xenotransplants, display higher degrees of tumour complexity, but are limited by inter-species differences in immunological mechanisms and chemokine signalling, compared to human cancer (48).

The possibility to easily generate primary tissue-derived organoids provides the basis for personalised *ex vivo* models, particularly in cancer research. Cancer-derived organoids display several traits that are similar to the original tumour and therefore allow the understanding of cancer biology by large-scale tumour bio-banking and high-throughput drug screenings, which gave rise to the discovery of novel anti-tumour compounds (49).

The availability of CRISPR/Cas9 systems enabled genetic engineering on (cancer) organoids with, however, highly variable efficiency of genome editing, depending on the route of CRISPR-Cas9 installation (50). As a proof of concept, sequential introduction of hallmark colorectal cancer mutations into human colonic organoids licenced growth factor independent proliferation of the organoids and mimicked aspects of the classical adenoma-carcinoma sequence (51). In the same study, re-implantation of these transformed organoids in a murine metastasis model demonstrated that, beside canonical cancer mutations, additional molecular lesions as chromosomal instability are necessary for an invasive cancer behaviour. Another example from the same group is the generation and usage of CRISPR/Cas9-guided knock-in of LGR5- and KRT20-reporters into cancer organoids to trace tumour stem cell behaviour *in vivo* (52).

On an individualised level, patient cancer-derived organoids (PDO) are suitable in the prediction of therapy response of an individual tumour or metastases by high-throughput screenings of therapies (e.g. compound screens, chemotherapy, irradiation) on a small specimen in neo-adjuvant settings (53, 54).

To depict the complexity of tumour microenvironments, including immuno-oncological cross-talks, distribution of drugs and metabolites in the tumour etc., the need for cancer organoid models exceeding the pure epithelial tissue was recently defined (55). The following two strategies are pursued and include the tumour epithelium and tumour-associated cell types such as, immune cells or stroma:

- The cultivation of organotypic tumour spheroids or tumour cells on air-liquid interfaces is a more holistic approach and mimics the complete tumour, however it requires a more substantial (mostly surgical) specimen, which excludes the use for neo-adjuvant strategies.
- Co-culture systems of PDO with separately sampled cells of the microenvironment benefit from pre-established PDO culture protocols. These models have the advantage that specific cellular components of the microenvironment can be addressed, and only small tumour specimens are necessary for PDO generation.

Since the need for these complex PDO models and the strategies to address the microenvironment are rather new, there are some promising reports indicating that establishing complex PDO models harbouring different cellular compartments are feasible and representative for the original tumour (53, 56–59)

The advances of organoid research in both cancer and inflammation (non-malignant) gave rise to potential model systems, which, for instance, could depict the transition from chronic inflammation to inflammatory-driven carcinogenesis.

## Standardisation Remains the Major Challenge for *In Vitro* Inflammation Models

As outlined, enormous advances in the stem cell and organoid field have emerged and their potential in translational research, and even healthcare, is obvious; however, current limitations of organoids remain an important caveat (**Figure 2B**).

A general limitation of organoid derivation is the high variability of the phenotypes that they can produce. Organoid and stem cell culture systems and their molecular outcome are critically dependent on the quality and properties of the sampled tissue. Variations in the pre-processing phase heavily interfere with immune signatures of these samples. Moreover, inflamed tissue is more likely to undergo cell death, might have different nutrient and culturing needs and thus the recovery rate of vital organoids from inflamed tissue is significantly lower (60). Whilst inflammatory signatures *ex vivo* are similar to their *in vivo* origin (9), particularly complex diseases such as IBD are characterised by a diversity of inflammatory flavours. Furthermore, biopsies are reflecting the biology of the sampled region, and therefore stem cells/organoids should be harvested from different regions. When using iPSCs, variations exist in organoids depending on the genetic background of the individual and the culture protocol used by the lab. Isogenic lines generated *via* gene-editing approaches can limit this variability in regard to genetic differences.

Because organoids from iPSCs are formed through differentiation of a homogeneous population, tissue-specific cell types and their microenvironment must be newly created. This challenges the use of iPSCs and despite significant similarities in structure and function between organoid and adult tissue, organoids often retain immature characteristics, making them more similar to foetal tissue. In an example of iPSC-derived intestinal organoid, this limitation could be resolved by either co-culturing it with T cells providing a more realistic growth niche (61), engineering of more sophisticated 3D scaffolds to improve organoid architecture and thus increase the similarity to the original tissue *in vivo* (62, 63), or *in vivo* transplantation, which resulted in mature intestinal epithelium with preserved intestinal stem cell niches, crypt/villus architecture and a laminated human mesenchyme, both supported by mouse vascular ingrowth (64). Apart from immature characteristics, organoids often show a limited lifespan once a certain size is reached. Due to the lack of diffusion, cells are not supplied with sufficient nutrients to support continued development. The use of bioreactors could improve the nutrient supply. Ideally, organoids are engineered towards the induction of endothelium development resulting in vascularization as shown recently (41). Another strategy would be to integrate endothelial cells, or their progenitors, during organoid development, and to include bioprinting methods to design 3D-scaffolds for the endothelial cells (65, 66). However, the original material tissue of iPSC-derived organoids is usually not inflamed, e.g. skin fibroblasts, and have been reprogrammed and cultured for many weeks to months in the absence of an inflammatory milieu. For the study of complex, multifactorial inflammation, iPSC-derived systems are therefore not the first choice in terms of a model. Vice versa, iPSC-derived organoids might be excellent tools to study mainly genetically driven inflammatory disorders, such as monogenic IBD.

Another important challenge is the magnitude of effort, time and expenses spent on organoid cultures. Whilst rather simple protocols are available for conventional organoid cultures at manageable costs, differentiation protocols include either expensive factors or culturing media or require conditioned media, which need to be produced by feeder cell lines. Co-culturing systems often include 2D models, which require much higher culturing efforts and time. Adding to this challenge, the generation of iPSC and their differentiation into specific tissue often require several weeks of intense culturing effort, which increases the risk of adverse events, e.g. contamination or undesired differentiation.

To combat the aforementioned issues, close collaboration of academic research and high-tech industrial partners may be a promising strategy to overcome the infrastructural challenges. Whilst academia can provide problem-derived ideas and a hypothesis-driven vision of novel disease models, industry can deliver the necessary technology, capacities for large-scale production and standardisation. Setting up these collaborative infrastructures are necessary and can foster future advances of stem cell technology.

## Conclusions and Future Perspectives

Organoids have been shown to keep key multicellular, anatomical and to some degree even functional hallmarks of real organs. In order to exploit these improved disease models, the application of high-throughput analysis techniques and large-scale perturbation tools to organoids is required. Single-cell multi-omics (19, 20) and imaging technologies can provide insight into underlying pathological mechanisms at different regulatory molecular layers. Physiological interactions of different organs could be modelled by using microfluidics and organ-on-a-chip technologies to study key systemic interactions in diseases. The combination of advanced organoid models and these techniques might even enable to understand the early molecular mechanisms that cause cells to deviate from a healthy to a disease trajectory (67). This might lead to the detection of biomarkers for the prodromal disease state and the identification of new drug targets to intercept diseases before manifestation of symptoms.

One of the visions of stem cell research is the development of stem-cell-derived regenerative tissue for engraftment and transplantation purposes. Recently, a group successfully engrafted human iPSC-derived kidney organoids into immunodeficient NOD/SCID mice, which reached a higher degree of maturation compared to *in vitro* kidney organoids but still markedly immature compared to the neighbouring mouse kidney tissue (68). Besides tissue repair, this technology can be a promising vehicle for targeted gene therapy, especially for monogenic disorders. The advances in CRISPR/Cas9 technology already allow selective genome editing with a reduced number of off-target effects by using engineered secondary RNA structures (69) or by implementing prime editing guide RNA that specifies the target site and encodes the desired edit (70). However, only complete and definite exclusion of any off-target DNA alterations are acceptable for transplantation purposes.

It will be of great importance to implement organoids as a platform for screening and testing personalised medicine treatments, as they are cultures of primary patient material. Whilst this is already possible for cancer (54), it will in the future also be possible for inflammatory disorders that affect epithelial tissues. We expect implementation of these systems in drug discovery, therapy guidance and tissue regenerative medicine. Therefore, joint efforts of academia and industrial partners are mandatory to surpass challenges in regard to safety and reliability.

## Author Contributions

All authors contributed to the discussion at the symposium workshop. The manuscript was conceptualised by FT, CK, AA, SI, PR, and PS, whilst FT coordinated the manuscript writing. The figures and tables were designed by FT, AA, and PS. All authors contributed to the article and approved the submitted version.

## Funding

This work was supported by the German Research Foundation (PR, PS, and CK: ExC 2167 “Precision Medicine in Inflammation”, AA: project no. 414216991, PS and CK: FOR 2488, PR: CRC1182) and the EU Innovative Medicine Initiative 2 Joint Undertaking (PR: “3TR”, grant agreement no. 831434).

## Conflict of Interest

The authors declare that the research was conducted in the absence of any commercial or financial relationships that could be construed as a potential conflict of interest.

The handling editor declared a shared affiliation, though no other collaboration, with several of the authors, CK, EK, and PS.
